# Supplementary material for: Materials tactile logic via innervated soft thermochromic elastomers
Source: Nat Commun. 2019 Sep 13;10:4187. doi: 10.1038/s41467-019-12161-1 (PMC6744443; doi:10.1038/s41467-019-12161-1)
Supplement: Supplementary file 1 — Supporting Information [file 41467_2019_12161_MOESM1_ESM.pdf]

Supplementary Information for

Materials Tactile Logic via Innervated Soft Thermochromic Elastomers, Jin et al., Nature Communications

Supplementary Notes

Supplementary Note 1. Modeling. We present numerically and graphically simulated data obtained using Comsol 4.2 Multiphysics (R) software using Joule heating physics. The geometry of the model is drawn in 3D based on the experimental parameters. The meshing was done using tetrahedral elements. The convective cooling and surface-radiation heat-transfer are applied to all surfaces as boundary conditions, but the bottom surface of the device is kept at constant temperature (room temperature). In all simulation electrical current are only passing through the center of the device (channel filled with EGaIn).

Supplementary Note 2. Shunt Circuits. We fabricated a parallel circuit with ten parallel shunts. We applied 1A current through this parallel circuit. All the shunts distribute the current flow evenly; that is, each shunt carries 0.1A, which is insufficient to induce color change in this system. Applying pressure to a portion of the shunt redistributes the current so that the "open' shunts receive more current; thus, the response is "distal" (i.e. away from the region being touched). To demonstrate this concept, we pressed seven shunts to redirect the current to the other three metal wires. In this case, the current in the 'open' shunts increases to 0.33 A per trace, which is sufficient to change the local color to blue. Likewise, pressing nine shunts redirects the current through the one remaining 'open' shunt, which creates sufficient current to change the local color to white. The interesting aspect of this concept is that the color change occurs away from the region being touched and does so without any central processor.

Supplementary Note 3. Thermochromic mechanism. We utilized commercial microcapsule pigments in our studies. We offer some insight into how these pigments change color with

temperature. The three components of these leuco pigments include fluorane dye as a color former, weak acids as the developer, and alcohols as the solvent[1]. These can be mixed to form a reversible thermochromic system (in our case, they were already combined into a pigment powder)[2]. The color change is driven by interactions between these components. The color former is an electron donating compound. The developer is an electron acceptor (proton donor) compound such as bisphenol A and phenols[3]. Typically, the colored complex of a color former and developer prevail below the melting point of the solvent. Color change always takes place at the melting temperature of the solvent[4]. When the solvent melts, the dye-developer complex is destroyed, and the system acquires the natural color of fluorane dye[5]. Such a reversible thermochromic system can be encapsulated in a polymer envelope to form a microcapsule as a pigment[6]. This microcapsule enables the solid-liquid thermochromic system to stay as a solid powder during heating, which can be directly blended with a polymer (here, PDMS).

Supplementary Figures

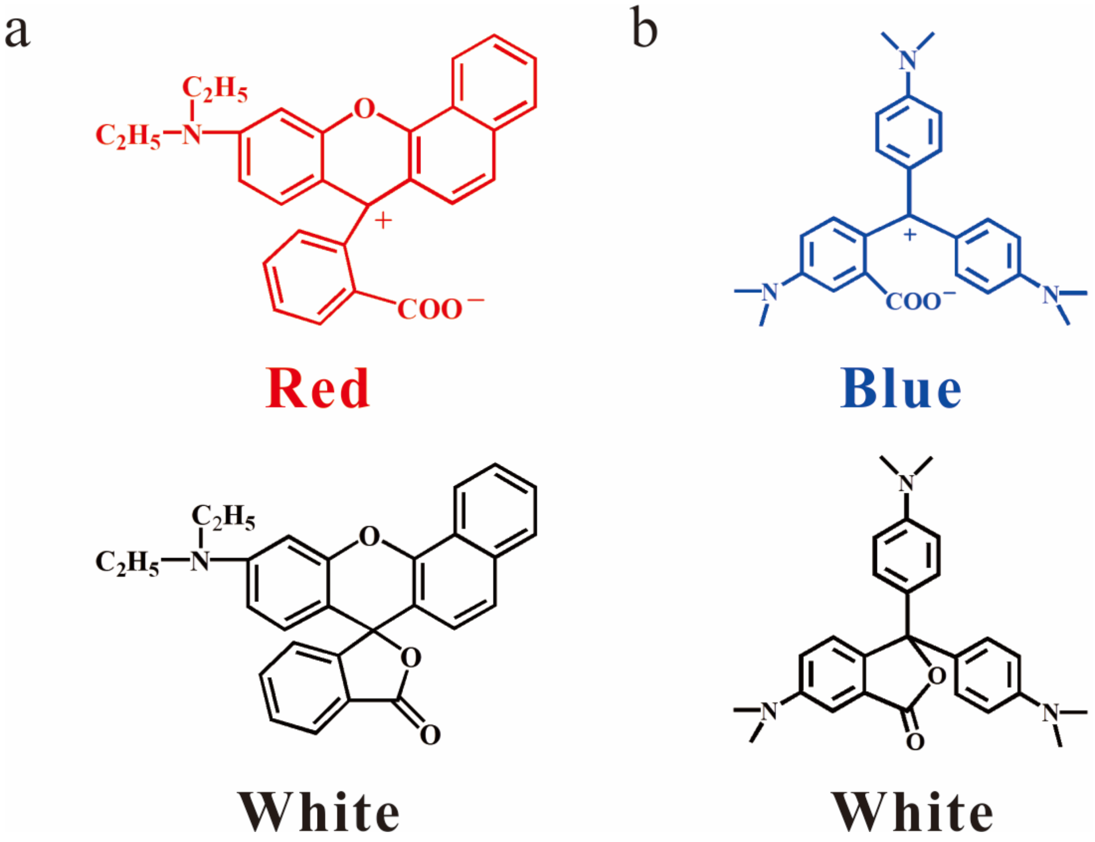

Supplementary Figure 1. **The molecular structures of leuco dyes within the pigment.** **a** Heat (Pressure) Rose Red TF-R1 dye. **b** Crystal Violet Lactone dye.

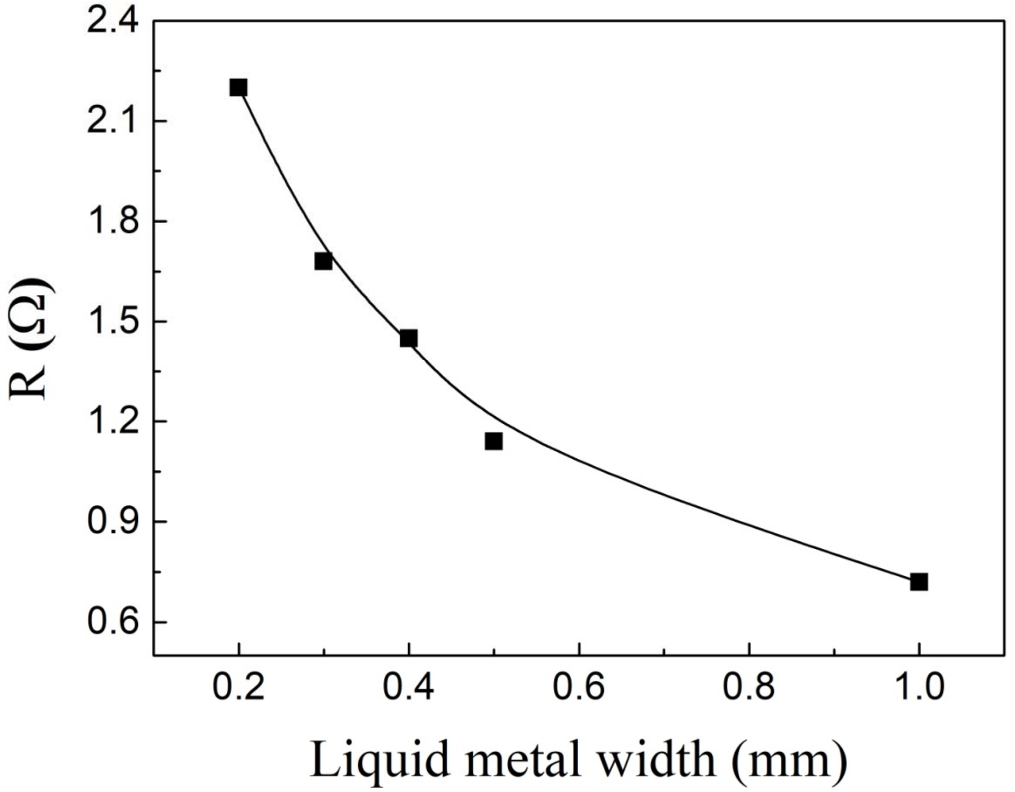

Supplementary Figure 2. Resistance of liquid metal versus microchannel width. Higher resistance give rise to higher power for constant current.

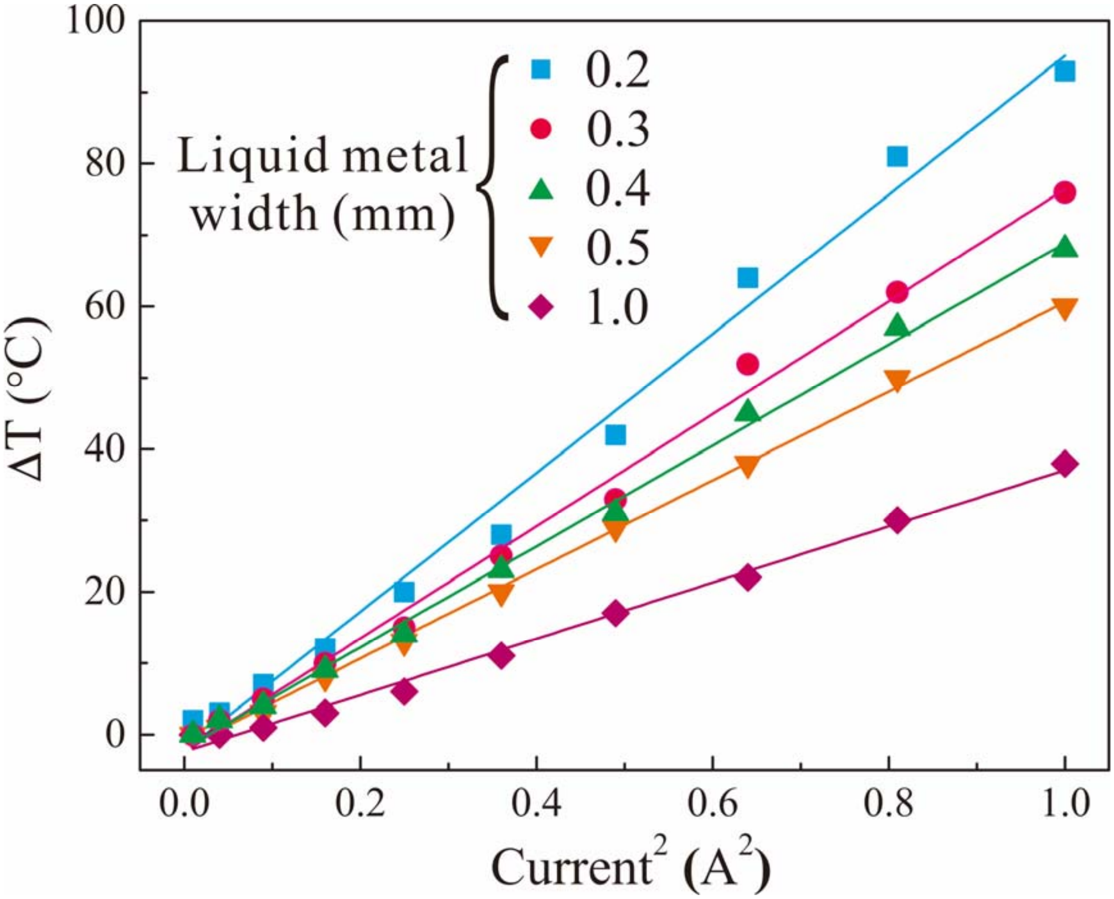

Supplementary Figure 3. Plot of temperature versus current squared The lines are best fits of the experimental data. (ΔT is surface temperature minus room temperature). The liquid metal in these devices are 50 mm long, 0.05mm tall. The widths refer to the widths of the microfluidic channels.

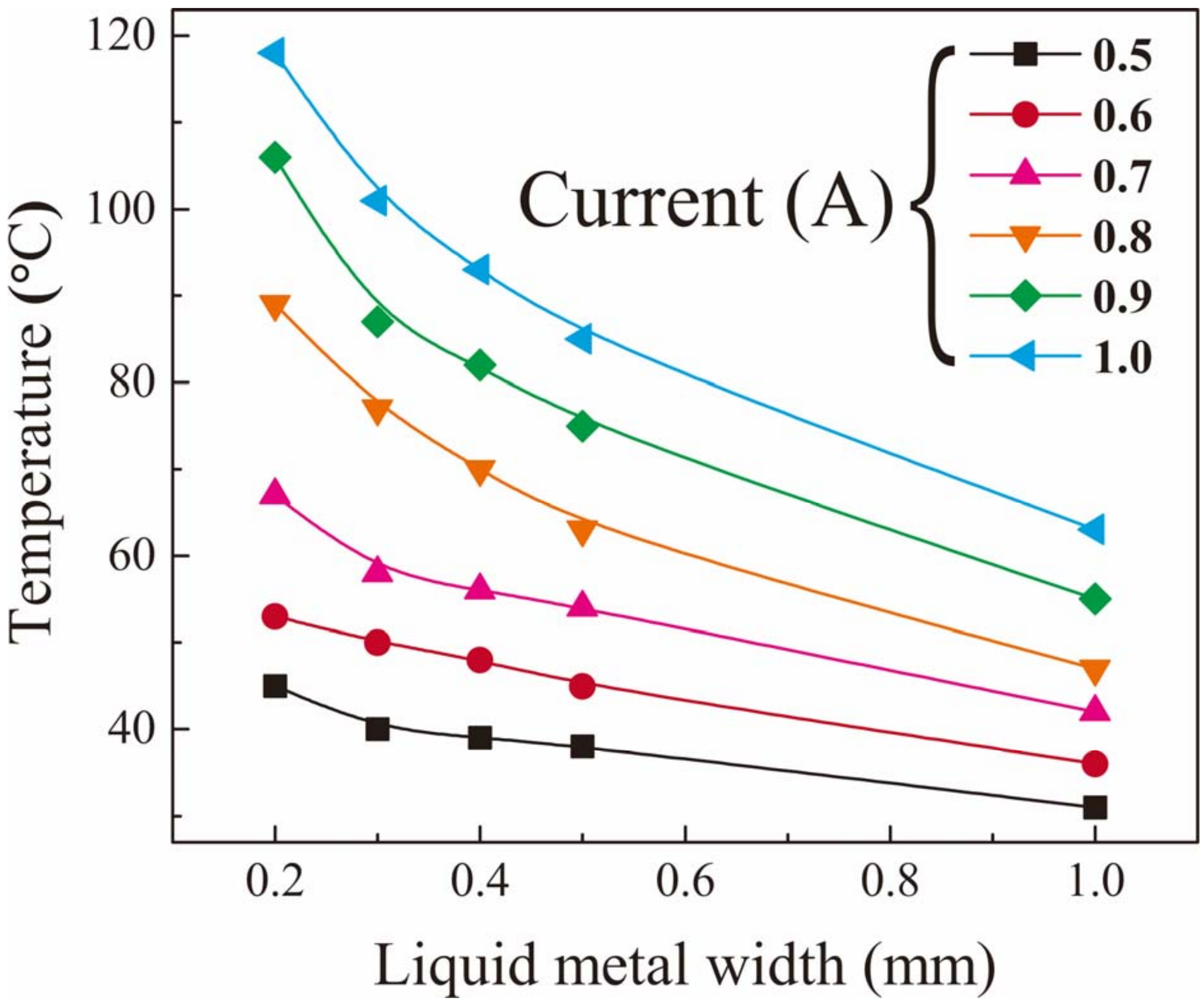

Supplementary Figure 4. Temperature of silicone as a function of geometry and current. We applied current on devices and recorded the highest surface temperature of the silicone slab at steady state. A plot of temperature versus width of the liquid metal wires is shown. The lines are shown to guide the eye. The numbers in the inset show the current applied to liquid metal wire (units of A). Wider channels of liquid metal had less resistance, and therefor did not heat as much as narrower liquid metal channels. Increasing the current increased the temperature, as expected.

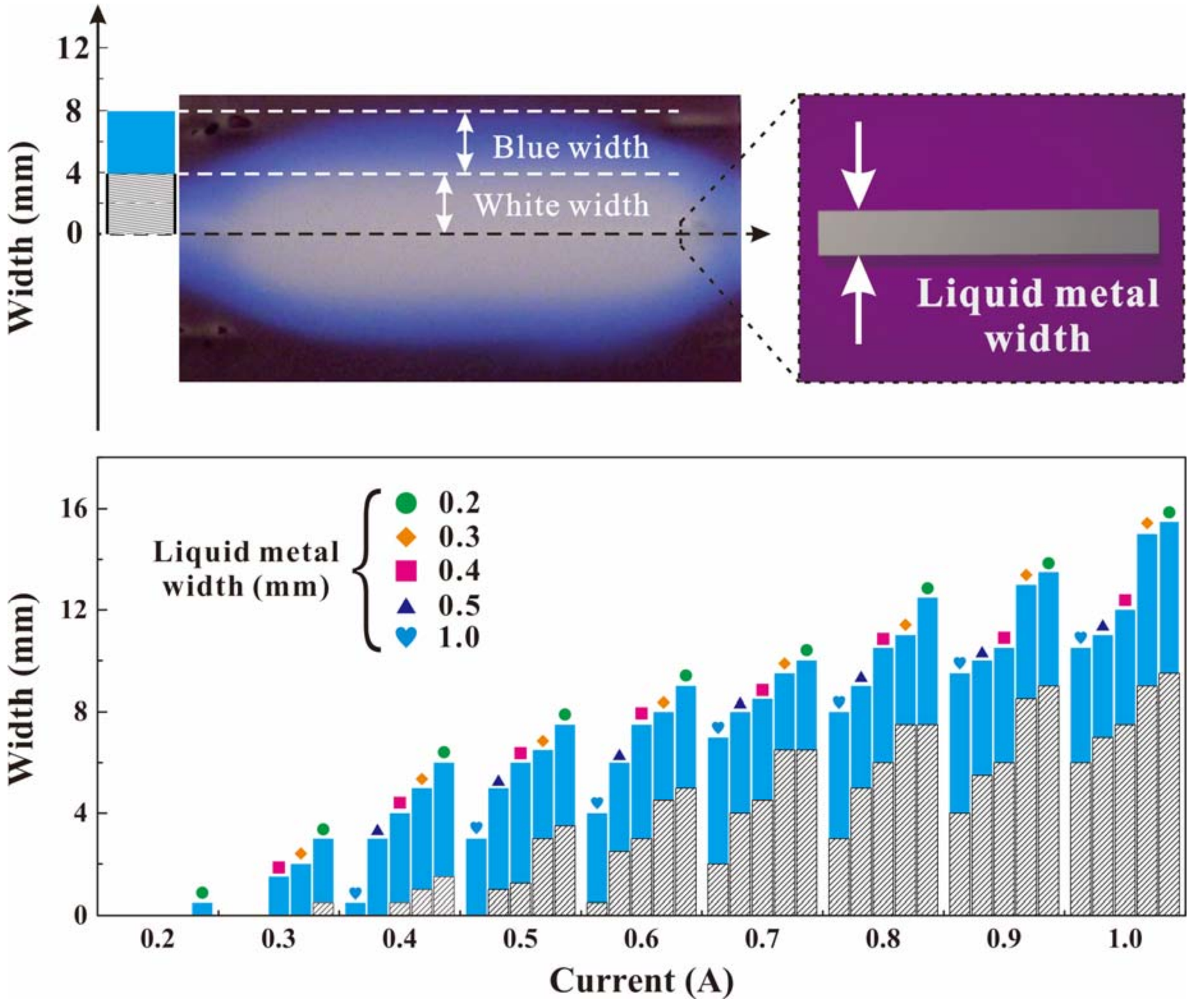

Supplementary Figure 5. Width of the color modulated area for different applied currents. The blue and white bars represent the width of the blue and white regions in the device, respectively. The silicone is initially purple due to the presence of both red and blue pigments. Heating causes the red to disappear first, followed by the blue with additional heating. As a result, the hottest regions appear white. The top image is an experimental image that shows the surface of a silicone slab with three regions of color (white = absence of blue and red, blue = absence of red, and purple = red + blue) along with a schematic of the relative width of the metal within the silicone. The average thickness of the PDMS in the device is 3.01 mm. The color layer is 1.21 mm thick and the clear layer is 1.80 mm.

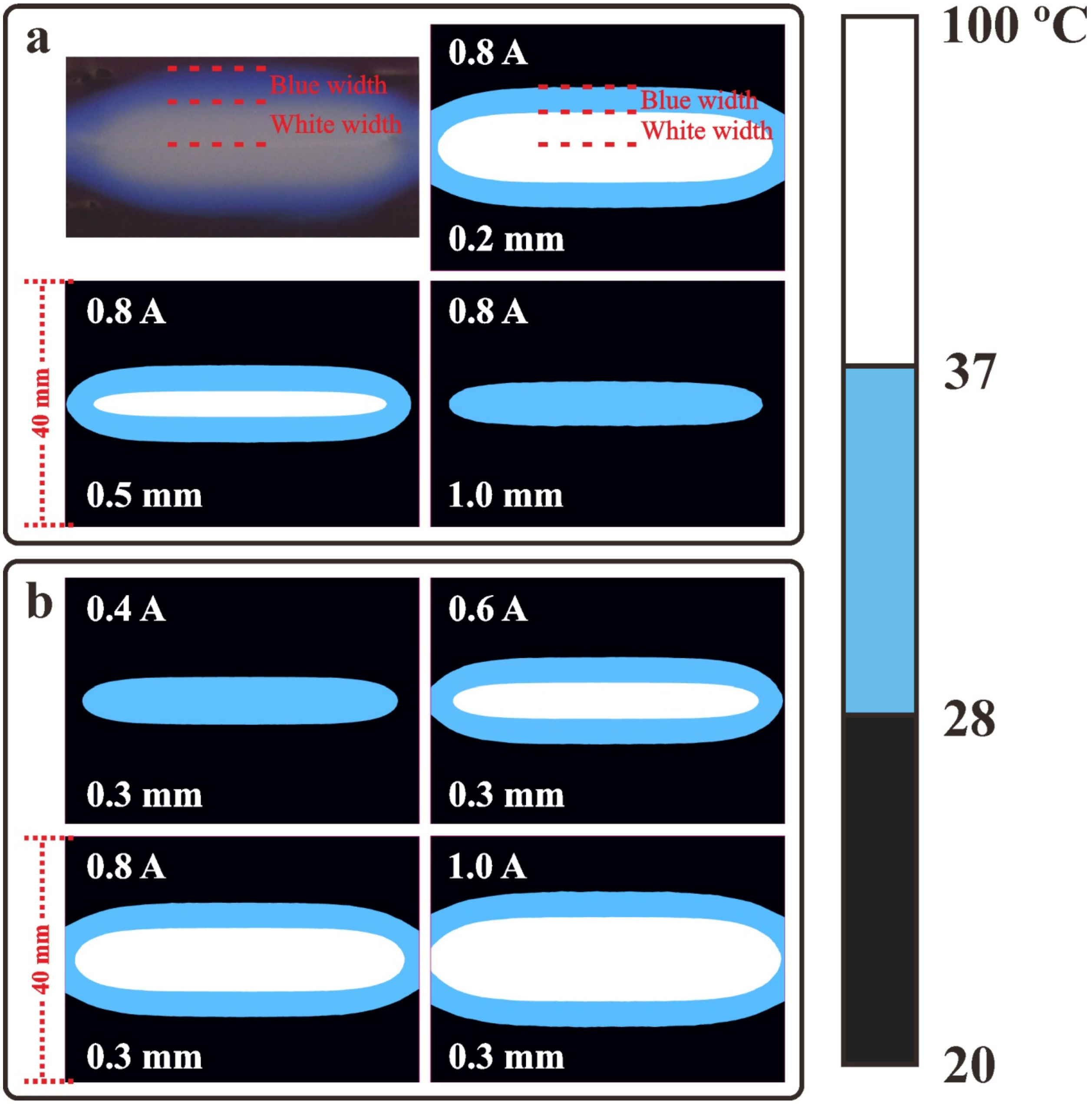

Supplementary Figure 6. 2D temperature profile obtained via simulation. Top down representation of the surface of the silicone slab featuring blue and red pigments, which changes temperature (and thus, color) in response to Joule heating. **(a)** Each simulation has the same applied current (A= Amps) but a different width of liquid metal wire as listed in each frame. **(b)** Each simulation has the same width of liquid metal wire but a different applied current.

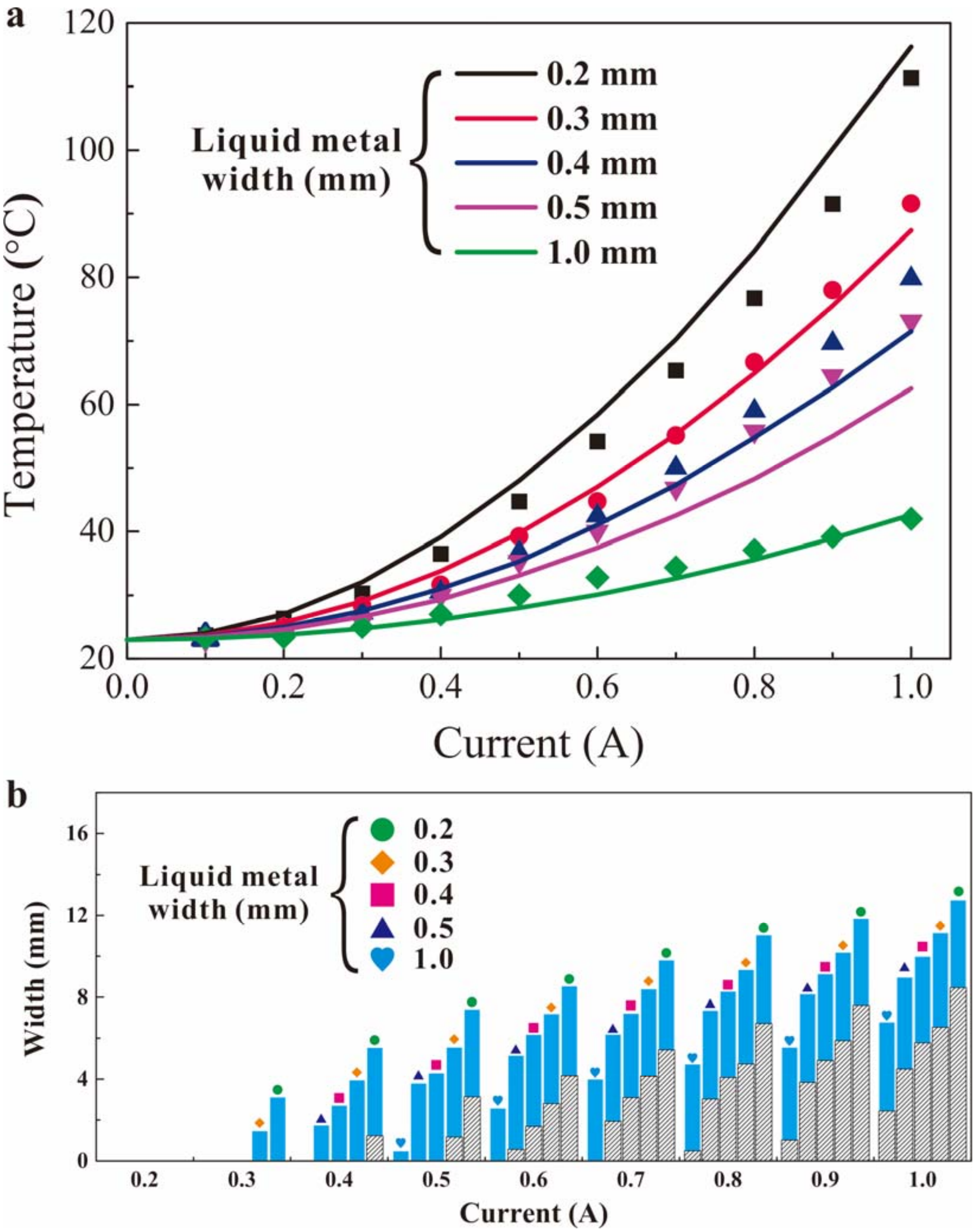

Supplementary Figure 7. Temperature versus current via Joule heating. a Comparison of simulated surface temperature with experimental data. The dots represent the experimental data while the lines represent the simulated data. **b** The simulated width of the color changed area versus the applied current. The blue and white bars represent the blue and white color width in the device, respectively.

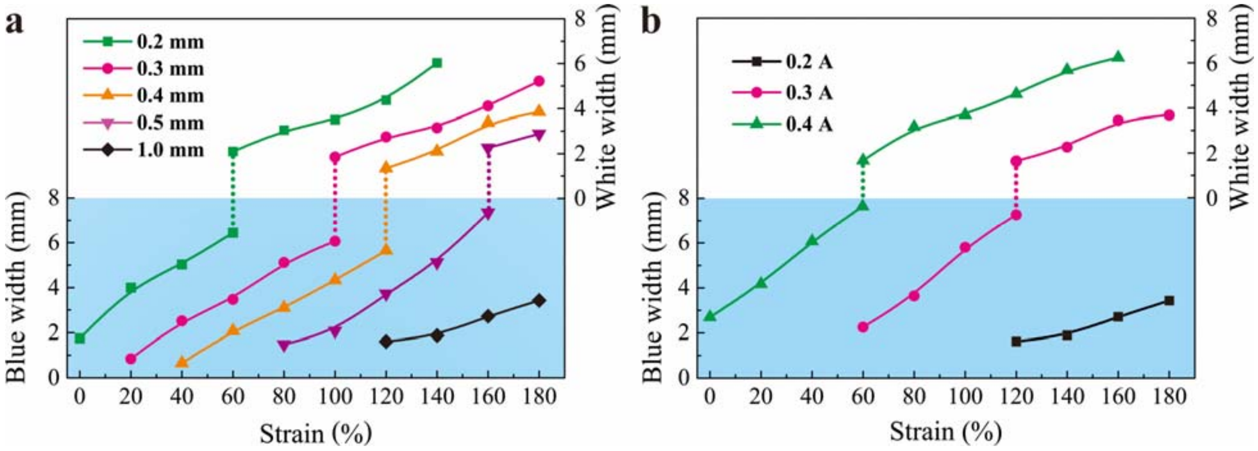

Supplementary Figure 8. Experimental measurement of the width of the color change. a The width of the color change region changes when stretched while applying 0.2 A. The legend lists the initial widths of the wires. **b** The width of the color change region (1.0 mm wide wire) changes when stretched as a function of current.

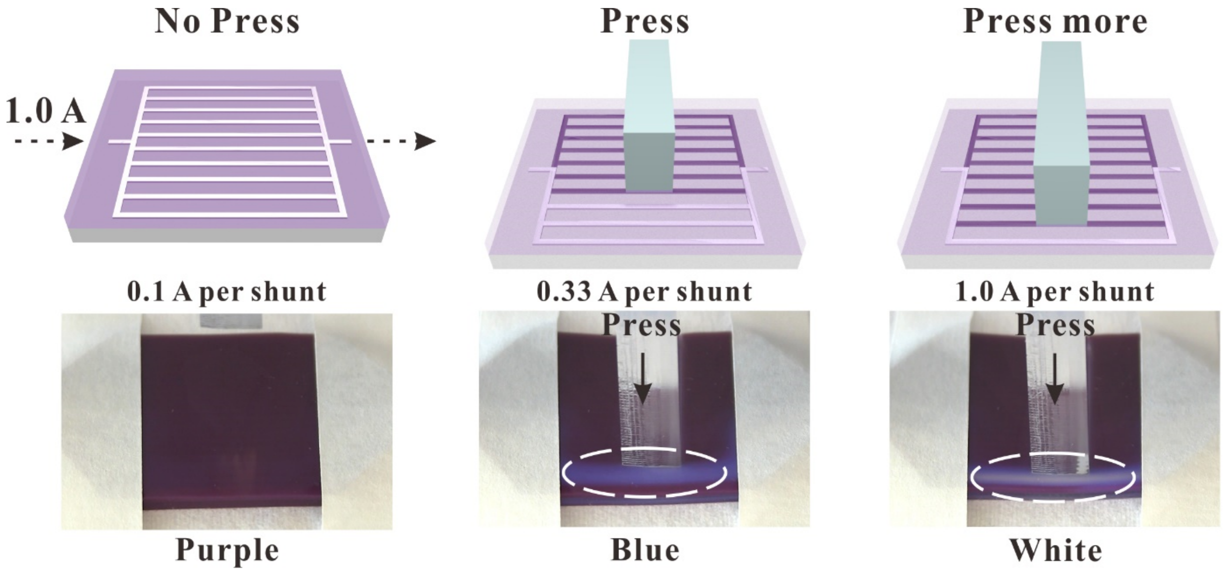

Supplementary Figure 9. Principle of touch-responsive liquid metal networks. Pressing on a portion of a parallel circuit with constant current redirects the current through the undisturbed paths.

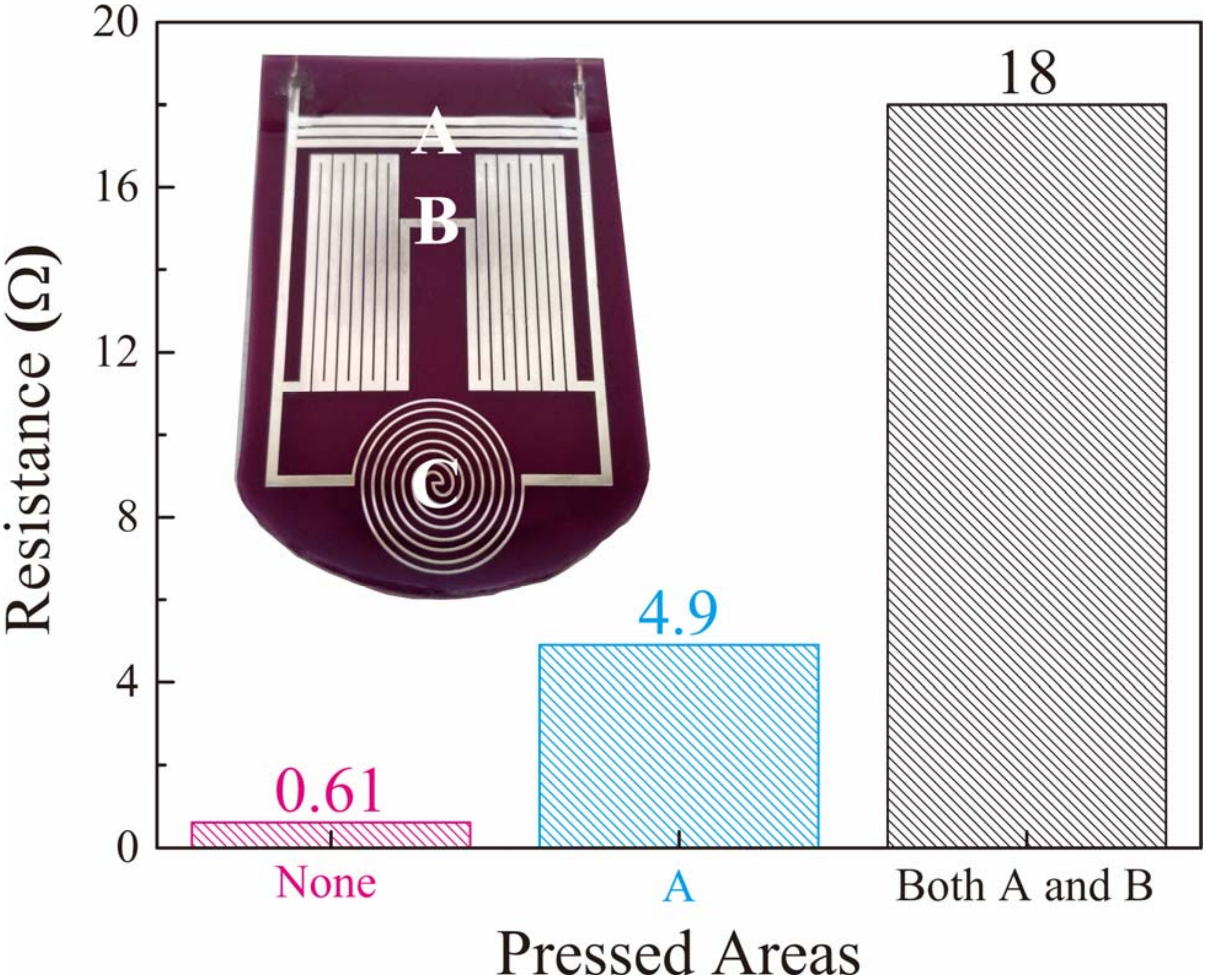

Supplementary Figure 10. Resistance of logic device versus input pressure signals. We pressed different regions and measured the resistance of the soft tactile logic device (shown in inset).

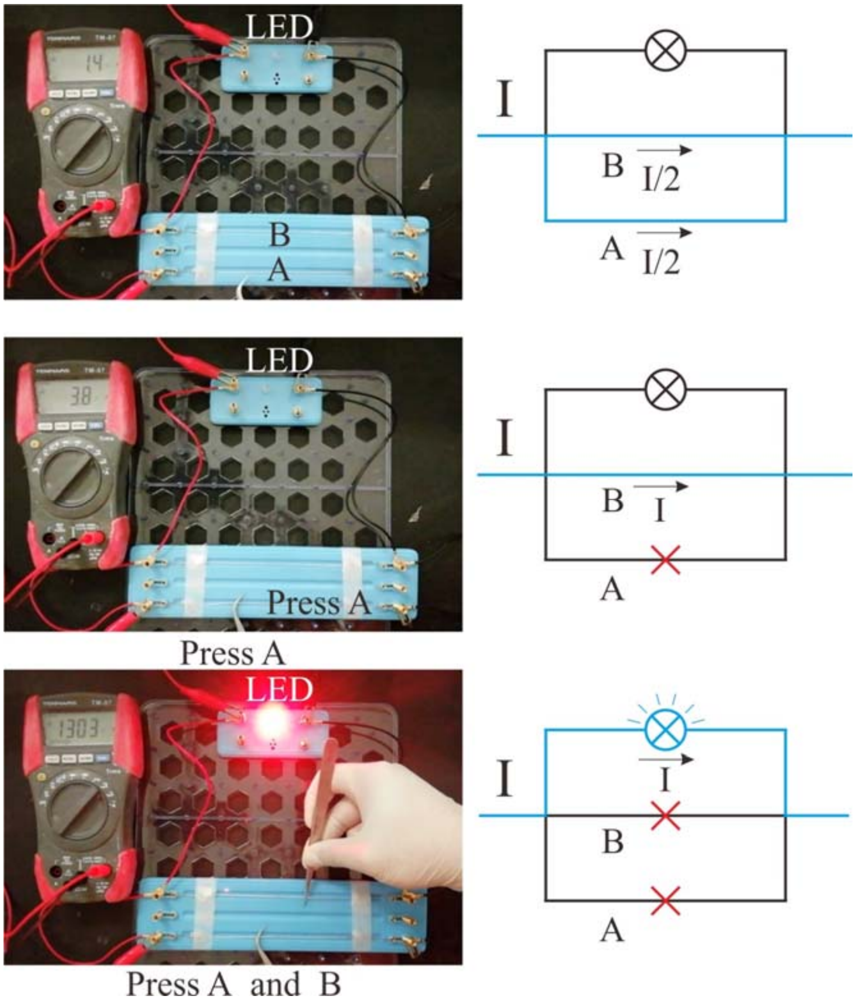

Supplementary Figure 11. Materials tactile logic using an LED as the output. We designed a circuit with three pathways (see Movie 10). Two of pathways consist of hollow silicone fibers filled with liquid metal (labeled as A and B). Another pathway includes a LED. We applied 0.02 A current to this circuit. When both of A and B are compressed, the LED can get the necessary potential to turn on. Although the entire demonstration here is not soft, the tactile portions are soft. This device demonstrates the ability of the concept of 'soft tactile logic' to extend beyond Joule heating and be useful for electronics.

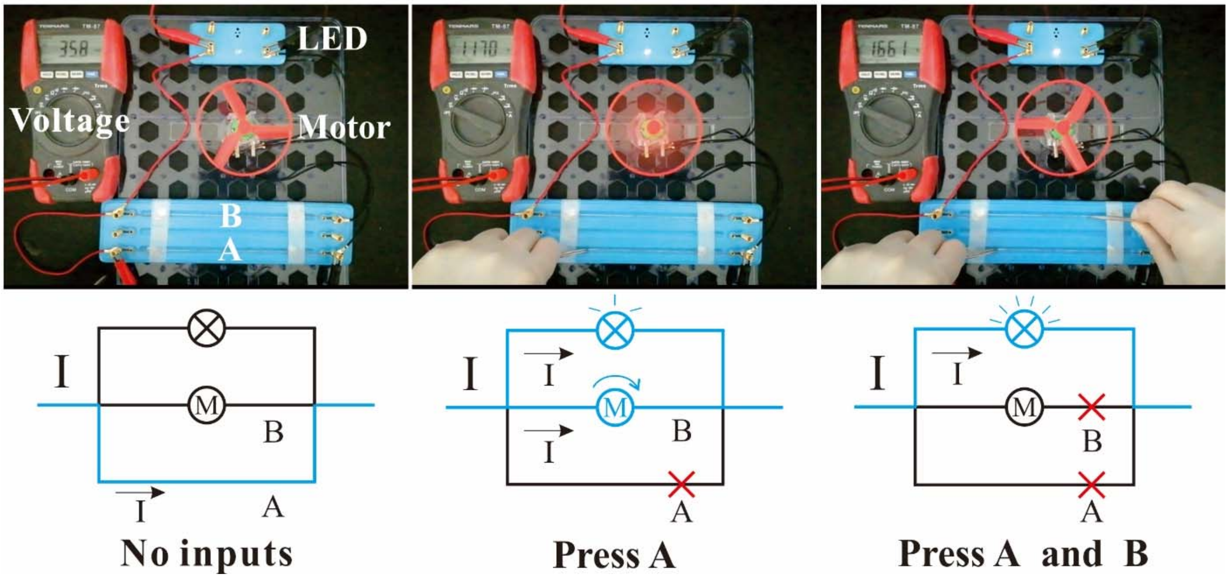

Supplementary Figure 12. Materials tactile logic using a motor as the output. Demonstration of a soft tactile circuit that has both a mechanical (fan) and electrical (LED) response. Region A and B consistent of silicone tubing with liquid metal inside the core. Compressing region A turns on the fan and the LED. When region A and B are pressed, the LED remains on and the fan turns off. See Movie 11.

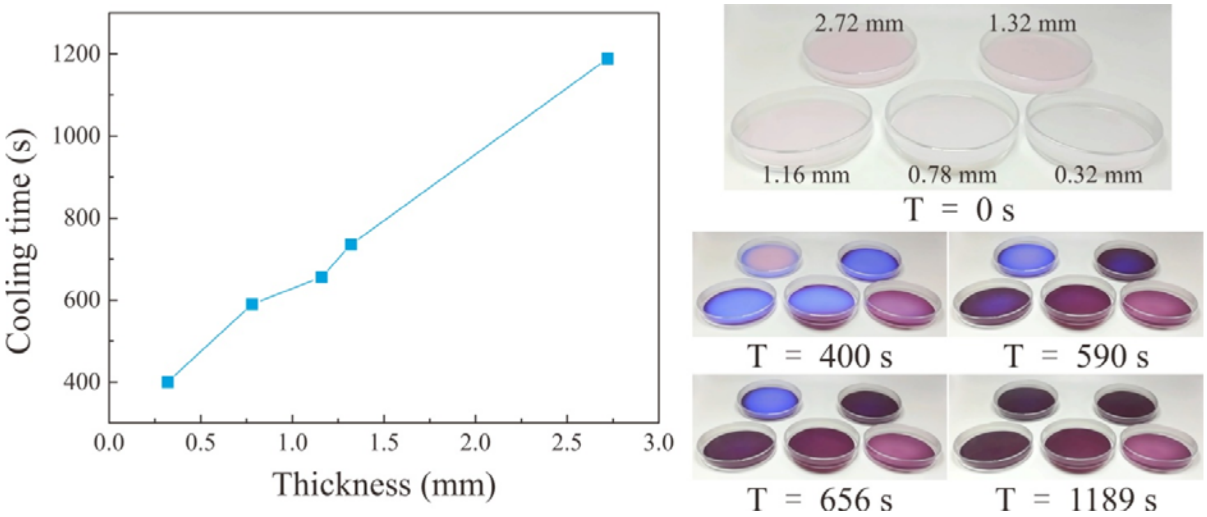

Supplementary Figure 13. Cooling response time of silicone versus thickness. The cooling time of thermochromic elastomers with different thickness. See Movie 12.

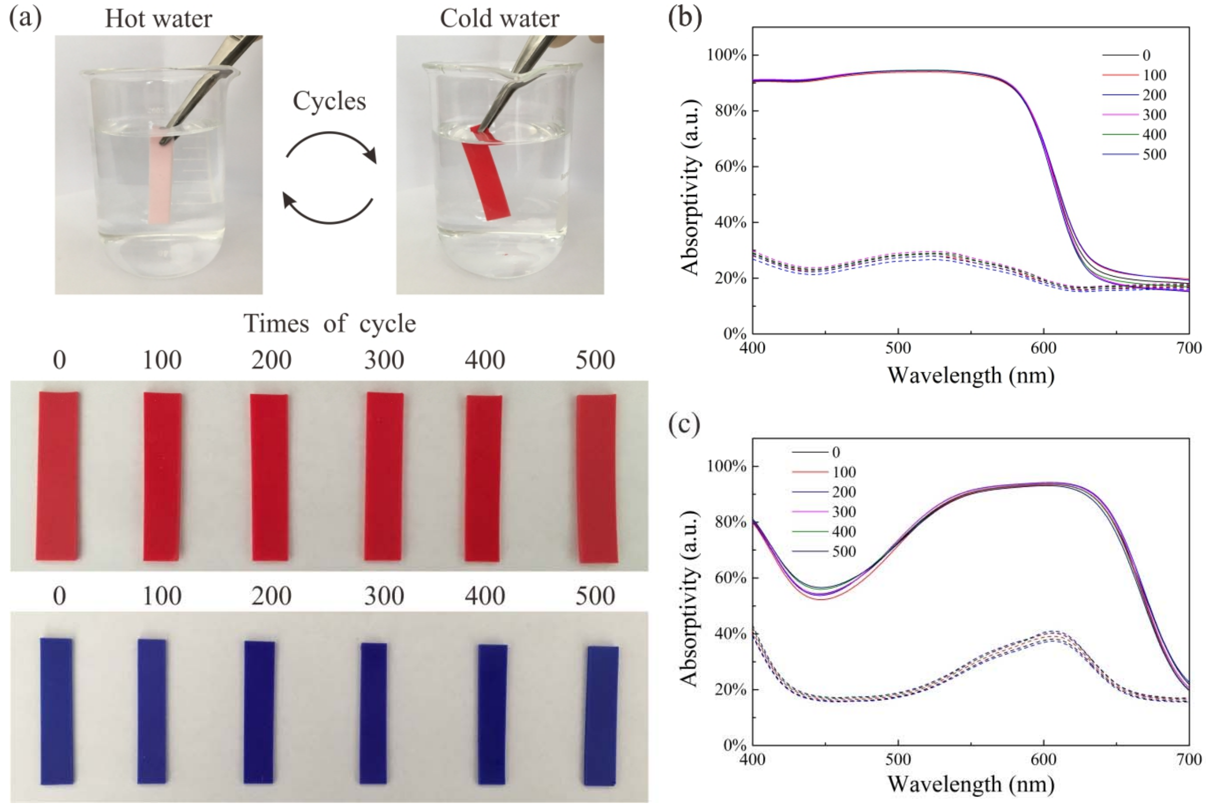

Supplementary Figure 14. Measurement of the color fastness of the pigments. **a** Color "fastness" test of thermochromic elastomers (using Slygard 184, Dow, as the silicone). We cycled the samples 500 times between hot and cold baths. There is no degradation in color during these cycles. To quantify the ageing of the pigment color, we report the absorptivity of the red and blue elastomers at different temperatures. **b** The absorptivity of red elastomers at 25°C (solid line) and 30°C (dotted line). **c** The absorptivity of blue elastomers at 25 °C(solid line) and 40 °C(dotted line).

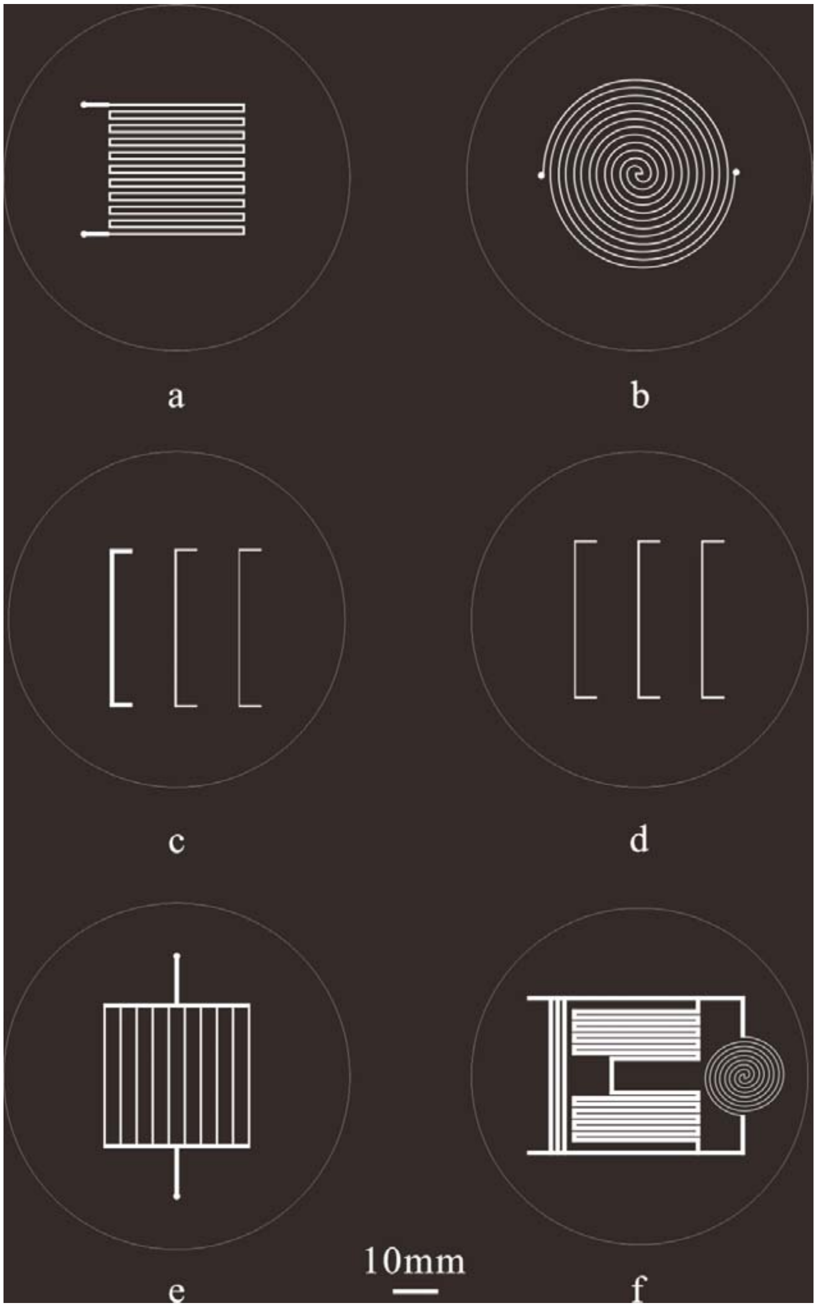

Supplementary Figure 15. The areal patterns of the liquid metal circuits used in this paper. **a** Pattern used for Figure 1f and Figure 2d. **b** Pattern used in Figure 1e. **c** and **d** Pattern used in Figure 2b. **e** Pattern used in Figure S8. **f** Pattern used in Figure 3.

Supplementary References

1. Panák, O., Držková, M. & Kaplanová, M. Insight into the evaluation of colour changes of leuco dye based thermochromic systems as a function of temperature. *Dyes Pigments* **120**, 279–287 (2015).
2. Jin, Y. *et al.* Preparation and luminescence studies of thermosensitive PAN luminous fiber based on the heat sensitive rose red TF-R1 thermochromic pigment. *Dyes Pigments* **139**, 693–700 (2017).
3. Panák, O., Držková, M., Kaplanová, M., Novak, U. & Klanjšek Gunde, M. The relation between colour and structural changes in thermochromic systems comprising crystal violet lactone, bisphenol A, and tetradecanol. *Dyes Pigments* **136**, 382–389 (2017).
4. MacLaren, D. C. & White, M. A. Competition between dye–developer and solvent–developer interactions in a reversible thermochromic system. *J Mater Chem* **13**, 1701–1704 (2003).
5. MacLaren, D. C. & White, M. A. Dye–developer interactions in the crystal violet lactone–lauryl gallate binary system: implications for thermochromism. *J Mater Chem* **13**, 1695–1700 (2003).
6. Jin, Y., Bai, Y., Zhu, Y., Li, X. & Ge, M. Thermosensitive luminous fiber based on reversible thermochromic crystal violet lactone pigment. *Dyes Pigments* **146**, 567–575 (2017).
