# Supplementary material for: Materials tactile logic via innervated soft thermochromic elastomers
Source: Nat Commun. 2019 Sep 13;10:4187. doi: 10.1038/s41467-019-12161-1 (PMC6744443; doi:10.1038/s41467-019-12161-1)
Supplement: Supplementary file 3 — Description of Additional Supplementary Files [file 41467_2019_12161_MOESM3_ESM.pdf]

Description of Additional Supplementary Files

File name: Supplementary Movie 1
Description: Soft Passive Displays. We applied 0.4A current on a simple device to show a passive display. Joule heating of the metal causes the regions above the metal to change color from red to white. The inset movie is the infrared (IR) temperature reading of the surface.

File name: Supplementary Movie 2
Description: Color Changing Silicone via Joule Heating. We injected liquid metal into a serpentine circuit and applied 0.7A current. The blue thermochromic within the silicone completely changes color to white in response to electrical current via Joule heating.

File name: Supplementary Movie 3
Description: Straight Microchannel Changes Color with Current. We applied 0.1-1.0 A current on a 0.4 mm wide, straight liquid metal microchannel. The device changed color dynamically in response to different currents.

File name: Supplementary Movie 4
Description: Adaptive Coloration. Liquid metal was injected into a serpentine circuit within silicone to fabricate a device. Modulating applied current allows the device (shown in the center image) to change color to match the background (shown around the perimeter). The device was purple when current was off due to the presence of red and blue pigments. It changed to blue at 0.2A due to the "disappearance" of the red pigment and changed to white at 0.5A due to the "disappearance" of the blue pigment.

File name: Supplementary Movie 5
Description: Dynamic Camouflage. We blended pigments into the elastomer to create multi-colored camouflage devices that change color in response to applied current.

File name: Supplementary Movie 6
Description: Mechanochromic Device. Stretching liquid metal wires causes the geometry of the wires to change, and thus, the resistance to increase. Applying a constant current during elongation allows the current density, and thus heating, to increase with strain. We stretched a device while maintaining 0.2A constant current. The color response of the device can be tuned by varying the geometry of the channels.

File name: Supplementary Movie 7
Description: Pressure Responsive Device. We applied 0.1A current to a serpentine circuit in silicone and pressed it with 100, 200, 300, and 400 kPa pressures at intervals of 15 s over an area of 1x1 cm2. The device changed color locally where we applied pressure due to changes in the local resistance.

File name: Supplementary Movie 8

Description: Shunt Circuits Respond to Compression by Redirecting Current. We fabricated a parallel circuit with ten parallel shunts and applied 1A current through this parallel circuit. All the shunts distribute the current flow evenly; that is, each shunt carries 0.1A, which is insufficient to induce color change in this system. Applying pressure to a portion of the shunt redistributes the current so that the other shunts receive more current; thus, the response is "distal" (i.e. away from the region being touched). To demonstrate this concept, we pressed seven liquid wires to redirect the current to the other three liquid metal wires. In this case, the current in the distal shunts increases to 0.33 A per trace, which is sufficient to change the local color to blue. Likewise, pressing nine shunts redirects the current through the one remaining 'open' shunt, which creates sufficient current to change the local color to white.

File name: Supplementary Movie 9
Description: Soft Tactile Logic Evoking Color Change. We fabricated a circuit to demonstrate 'soft tactile logic'. This device consists of two "input" areas (labeled as A and B) and one "display" area (labeled as C). Here, the input signals are pressure. Similar to a threshold voltage in a transistor, there is a threshold pressure required to induce a sufficient signal to cause color change in the "display region". We applied 0.4 A current and input pressure signals to regions A and B. Region C responded to the analog input (pressure via touch) by changing the color between purple, blue, or white (a digital output).

File name: Supplementary Movie 10
Description: Soft Tactile Logic for Electronic Applications. We designed a parallel circuit with three pathways. Two of pathways consist of hollow silicone fibers filled with liquid metal (labeled as A and B). Another pathway includes a LED. We applied 0.02 A current to this circuit. When both of A and B are compressed, the LED receives the necessary potential to turn on. See Figure S10 for a circuit diagram.

File name: Supplementary Movie 11
Description: Soft Tactile Logic for Mechanical Applications. Demonstration of a soft tactile circuit that has both a mechanical (fan) and electrical (LED) response. Region A and B consistent of silicone tubing with liquid metal inside the core. Compressing region A turns on the fan and the LED. When region A and B are both pressed, the LED remains on and the fan turns off. See Figure S11 for a circuit diagram.

File name: Supplementary Movie 12
Description: Response Time of Color Change During Cooling. We prepared five thermochromic elastomers. The movie shows the color change as they cool to room temperature after removing them from a 60 °C oven. As expected, the thinner samples change color faster than the thicker samples.
